# Inosine as a Tool to Understand and Treat Central Nervous System Disorders: A Neglected Actor?

**DOI:** 10.3389/fnins.2021.703783

**Published:** 2021-08-24

**Authors:** Francisney Pinto Nascimento, Sérgio José Macedo-Júnior, Fernanda Rocha Lapa-Costa, Fernando Cezar-dos-Santos, Adair R. S. Santos

**Affiliations:** ^1^Programa de Pós-Graduação em Biociências, Laboratório de Neurofarmacologia Clínica, Faculdade de Medicina, Universidade Federal da Integração Latino-Americana, Foz do Iguaçu, Brazil; ^2^Centro de Inovação e Ensaios Pré-clínicos, Florianópolis, Brazil; ^3^Faculdade de Farmácia, Universidade do Contestado, Concórdia, Brazil; ^4^Programa de Pós-Graduação em Neurociências, Laboratório de Neurobiologia da Dor e Inflamação, Universidade Federal de Santa Catarina, Florianópolis, Brazil

**Keywords:** adenosine, uric acid, pain, depression, Alzheimer, Parkinson, memory, neural regeneration

## Abstract

Since the 1970s, when ATP was identified as a co-transmitter in sympathetic and parasympathetic nerves, it and its active metabolite adenosine have been considered relevant signaling molecules in biological and pathological processes in the central nervous system (CNS). Meanwhile, inosine, a naturally occurring purine nucleoside formed by adenosine breakdown, was considered an inert adenosine metabolite and remained a neglected actor on the purinergic signaling scene in the CNS. However, this scenario began to change in the 1980s. In the last four decades, an extensive group of shreds of evidence has supported the importance of mediated effects by inosine in the CNS. Also, inosine was identified as a natural trigger of adenosine receptors. This evidence has shed light on the therapeutic potential of inosine on disease processes involved in neurological and psychiatric disorders. Here, we highlight the clinical and preclinical studies investigating the involvement of inosine in chronic pain, schizophrenia, epilepsy, depression, anxiety, and in neural regeneration and neurodegenerative diseases, such as Parkinson and Alzheimer. Thus, we hope that this review will strengthen the knowledge and stimulate more studies about the effects promoted by inosine in neurological and psychiatric disorders.

## Introduction

**FIGURE 1:**
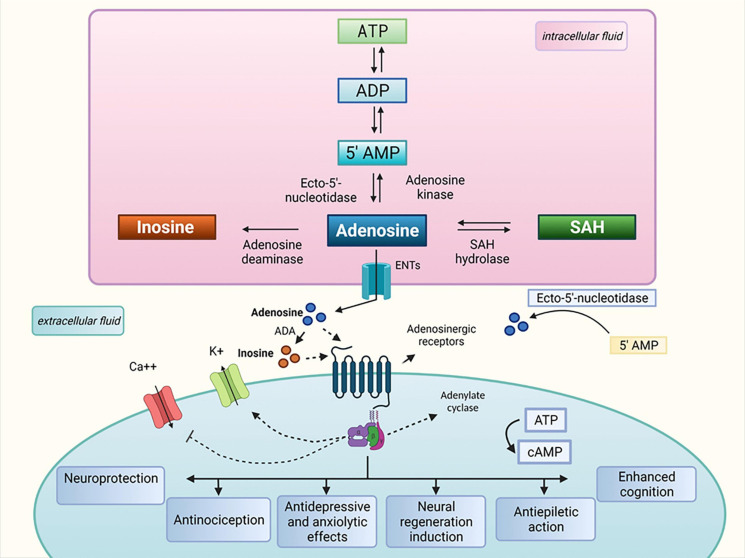
Inosine metabolism and proposed therapeutic properties. ATP, adenosine triphosphate; ADP, adenosine diphosphate; ADA, adenosine deaminase; AMP, adenosine monophosphate; Ca++, calcium; cAMP, cyclic adenosine monophosphate; ENTs, equilibrative nucleoside transporters; K+, potassium; SAH, *S*-adenosylhomocysteine.

The history of purine nucleosides began and gained therapeutic importance in 1929 with a paper published by Drury and Szent-Gyorgyi, who described the potent actions of purine on the heart and blood vessels, nucleotides and nucleosides, ATP, and adenosine (Burnstock, 2006). In the 1970s, the hypothesis that adenosine 5′-triphosphate (ATP) was a transmitter in non-adrenergic, non-cholinergic (NANC) inhibitory nerves was confirmed, and ATP was identified as a co-transmitter in sympathetic and parasympathetic nerves. The aforementioned supported the concept of purinergic neurotransmission and enhanced interest in the role of purine nucleosides in the brain and spinal cord, which was reported in various studies (North and Verkhratsky, 2006; Burnstock, 2007, 2020). In the subsequent decades, a number of studies demonstrated that adenosine can act as a signaling molecule essential for biological and pathological processes of the central nervous system. At the same time, evidence showed that adenosine metabolism generates inosine, a metabolite with possible biological and pharmacological effects in the peripheral and central nervous systems (see Figure 1). Inosine, which was first considered an inert adenosine metabolite after being used as a nutritional supplement to improve muscle function in trained endurance runners (Williams et al., 1990; McNaughton et al., 1999), has gained special attention due to a few studies in the 1990s that demonstrated that inosine is a signaling molecule that can modulate the immune system when produced under stressful conditions, such as those that occur during injury, ischemia, and inflammation. In these situations, an elevation in extracellular inosine concentrations due to high adenosine metabolism levels caused by an increase in adenosine deaminase expression was demonstrated (Haskó et al., 2000; Garcia Soriano et al., 2001; Liaudet et al., 2002; Eltzschig et al., 2006; Eltzschig, 2009; Fredholm et al., 2011).

The theory that inosine contributes to, or mediates adenosine effects is reinforced by studies that consider inosine as a natural trigger of adenosine receptors (Jin et al., 1997; Tilley et al., 2000; Gomez and Sitkovsky, 2003; Haskó et al., 2004; Eltzschig et al., 2006). These receptors were first formally recognized by Burnstock in 1978 and are subdivided into four subtypes: A_1_, A_2__*A*_, A_2__*B*_, and A_3_. The adenosine A_1_ and A_3_ receptors preferentially interact with members of the Gi/o family of G proteins, lowering the intracellular levels of cyclic adenosine monophosphate (cAMP), whereas the adenosine A_2__A_ and A_2__B_ receptors interact with members of the Gs family of G proteins, elevating intracellular cAMP (Ralevic and Burnstock, 1998; Eltzschig et al., 2006). Inosine binds to the adenosine receptors with affinities that are typically lower than that of adenosine. Although direct binding of inosine to adenosine A_2__B_ receptor has not been demonstrated, there is convincing evidence suggesting that inosine interacts functionally with all four adenosine receptor subtypes to elicit various effects depending on the species’ biological context (Nascimento et al., 2010; Kaster et al., 2013; Lapa et al., 2013; Muto et al., 2014; Doyle et al., 2017, 2018; Junqueira et al., 2017).

In the last four decades, a number of studies have shown that receptor-mediated inosine effects involve decreased inflammation, neuroprotection/neuroregeneration, and axon outgrowth in the central nervous system, which elicits scavenger activities and immunomodulatory effects. These effects directly reflect the therapeutic potential observed in pathological processes involved in chronic pain, schizophrenia, depression, anxiety, and neurodegenerative diseases such as Parkinson’s and Alzheimer’s disease (for a summary see Table 1). Given the increasing interest in the pharmacological potential of inosine as a new alternative therapy for CNS diseases, this review will focus on inosine effects in preclinical and clinical studies and describe the “state of the art” of the scientific research panorama regarding this promising molecule.

**TABLE 1:** Summary of *in vitro*, preclinical and clinical studies that have demonstrated the therapeutic potential of inosine to treat neurological and psychiatric diseases.

**CNS disorder**	**Specie**	**Agent**	**Dose/Concentration/Route**	**Effect**	**References**
Pain	Mice	Inosine	10–300 mg/kg/i.p. and p.o. 10 μg/i.c.v./i.t.	Inosine mediates reduction time of licking in the formalin test, inhibition of glutamate-induced nociception, reduction mechanical allodynia and hyperalgesia	Nascimento et al., 2010
Pain	Mice	Inosine	10 mg/kg/i.p.	Inosine promotes antinociception through pertussis toxin sensitive G-protein coupled receptors and voltage gated K+ channel, large conductance Ca2+-activated and ATP-sensitive K+ channels or inactivation of voltage-gated Ca2+ channels	Macedo-Junior et al., 2013
Pain	Mice	Inosine	10 and 100 mg/kg/i.p	Inosine reduces flinching behavior induced by formalin and systemic and central nociception in A1-wild type mice	Nascimento et al., 2015
Pain	Mice	Inosine	200–600 nmol/i.t.	Inosine inhibits nociceptive response induced by injection of capsaicin and increases ATP, ADP, AMP, adenosine, inosine, hypoxanthine, xanthine, and uric acid CSF levels	de Oliveira et al., 2016
Learning and memory	Rat	Inosine	100 and 200 mg/kg/i.p.	Learning and memory improvement, levels of TNF-alpha reduction, antioxidant properties increment	Ruhal and Dhingra, 2018
Epilepsy	Rat	Inosine	500 and 1000 mg/kg, i.p.	Inosine increases number and total time of spike-waves discharges	Kovács et al., 2015a, b
Epilepsy	Mice	Inosine	400 nmol/4 μl, i.c.v.	Inosine protect against quinolinic-acid induced seizures	Ganzella et al., 2011
Alzheimer	Rat	Inosine	100 mg/kg, i.c.v.	Inosine induces memory deficit prevention and AChE activity reduction	Teixeira et al., 2020
Parkinson	Dopaminergic cells culture	Inosine	—	H_2_O_2_-toxic effect on dopaminergic cells reduction, indicators of free radical generation and oxidative damage in MES 23.5-astrocytes co-cultures were reduced	Cipriani et al., 2010
Parkinson	Human	Inosine	500 mg; p.o.	Average serum urate increment (more pronounced in woman), reduction of motor symptoms in UPDRS scale	Schwarzschild et al., 2019
Neural regeneration	Goldfish	Inosine	50 and 100 μM	Inosine increases retinal ganglion cells neuronal outgrowth and GAP-43 levels	Petrausch et al., 2000
Neural regeneration	PC12 cells and neuronal embryonic cells Sprague-Dawley rat fetuses	Inosine	100 μM – 1 mM	Inosine activates Mst3b kinase activity	Irwin et al., 2006
Anxiety	Mice	Inosine	10 – 300 mg/kg, i.p.	Inosine antagonizes anxiety-reducing actions of benzodiazepines	Crawley et al., 1981
Anxiety	Mice	Inosine	10 – 60 mg/kg, s.c.	Inosine presents anxiolytic effect in the Vogel-type anti-conflict test	Okuyama et al., 1998
Depression	Mice	Inosine	10 mg/kg, i.p.	Inosine reduces mice immobility time in the forced swim test and in the tale suspension test	Kaster et al., 2013
Depression	Human	Inosine plasma levels	—	Inosine plasma levels are significantly higher in individuals treated with antidepressant drugs when compared to healthy individuals	Zhou et al., 2019

## Inosine and Pain

Adenosinergic and purinergic ligands are well-known modulators of acute and chronic pain of different etiologies in animals and humans both centrally and at the periphery, displaying beneficial and sometimes deleterious roles in pain pathology (Sawynok, 1998; Sawynok and Liu, 2003; Adebiyi et al., 2019). Most studies evaluating the analgesic effect of adenosine or adenosine receptor agonists have shown that these substances are able to enhance the pain threshold. However, in some cases these drugs can facilitate pain transmission. These controversial findings are probably due to the distinct signaling and distribution of adenosine receptors. Adenosine A_1_ and A_3_ receptors are coupled to inhibitory G proteins while the A_2__A_ and A_2__B_ are coupled to stimulatory G proteins. Further, the distribution of adenosine receptors in the periphery and central nervous system also are implicated in how these drugs act on pain modulation (Sawynok, 1998; Fredholm et al., 2011).

Reports detailing the antinociception – analgesia in pain animal models – exhibited by adenosine are not a novelty; however, until a few years ago, there were no studies evaluating the inosine antinociceptive effects. The first study that aimed to investigate the potential analgesic effects of inosine was published by our research group (Nascimento et al., 2010). This study used several acute pain animal models and demonstrated the antinociceptive action of this nucleoside in mice and rats. Inosine induced pain behavior reduction when given by oral, intraperitoneal, intratecal and intracerebroventricular, suggesting its systemic and central activity. Further, these findings suggest that inosine crosses the brain blood barrier (Nascimento et al., 2010). In addition, Nascimento and colleagues demonstrated that adenosine A_1_ and A_2__A_ receptors are involved in the inosine antinociceptive effects. Also, this same study evaluated that inosine was able to reduce nociception in chronic neuropathic and inflammatory pain models. It suggests that inosine acts on neural and inflammatory mechanisms to reduce pain threshold. Some years later our group using pharmacological, biochemical and genetic approaches confirmed that inosine antinociceptive action was dependent on the adenosine A_1_ receptor. Finally, this study proved that inosine is an endogenous agonist of adenosine A_1_ receptor (Nascimento et al., 2015).

In addition to the involvement of adenosine receptors in the antinociceptive effects of inosine, Macedo-Junior et al. (2013) showed that the inosine effects involves the activation of voltage gated K^+^ channels, large conductance Ca2^+^ and ATP-sensitive channels and the blockade of voltage-gated Ca2^+^ channels. The involvement of these ion channels could occur through adenosine A_1_ or A_2__A_ receptor activation. Peripherally, the inosine antinociception is mediated only by adenosine A_1_ receptors but not by A_2__A_ (Macedo-Júnior et al., 2021).

Apart from the inosine binding to adenosine A_1_ receptor to reduce pain, a study has shown that spinal administration of inosine increased the CFS levels of ATP, ADP, AMP, adenosine, hypoxanthine, xanthine, and uric acid (de Oliveira et al., 2016). These results suggest that inosine when reach the CNS could induce antinociceptive effect through these other nucleosides, because most of them have presented antinociceptive effects too (Sawynok and Liu, 2003).

Regarding the effects of inosine in humans, the clinical studies evaluating the involvement of inosine on pain are quite scarce. There are no clinical trial studies evaluating the analgesic effect of inosine in any kind of pain. However, there is some evidence that inosine could participate or be involved in clinical pain mechanisms. A study by Schmidt and coworkers found changes in extracellular inosine levels in patients experiencing pain. The authors suggest that shifts in neuronal and glial energy metabolism might be correlated with pain transmission mechanisms. CSF levels of inosine and other purines associated with ATP metabolism are significantly increased in chronic pain patients compared to control patients and are significantly correlated with pain intensity measured using a visual analog scale (Schmidt et al., 2010). Conversely, serum purine metabolites are increased in fibromyalgia patients (i.e., the dysregulation of pain pathways leading to central sensitization) compared to controls. In these patients, the magnitude of inosine detection was substantially higher than that of other metabolites (Fais et al., 2013).

Inosine is also a potential biomarker in plasma from patients experiencing non-traumatic chest pain or unstable angina due to potential acute cardiac ischemia/myocardial infarction (Farthing et al., 2007; Ameta et al., 2016). High levels of this nucleoside have already been reported in mouse hearts (Farthing et al., 2006) and in observational studies in humans (Al-Shamiri et al., 2009; Farthing et al., 2011). However, early evidence has shown that adenosine, but not inosine, may cause chest pain in healthy individuals. Then, the high levels of adenosine would be responsible for these inosine increments (Lagerqvist et al., 1990).

While the mechanisms mediated by inosine in nociceptive and neuropathic pain in animals have been studied in the last years and the results are encouraging, we need further studies to test and to better understand these actions. On the other hand, regarding clinical research on pain, there is a complete avenue to be explored and filled before we can say that this nucleoside could be useful in pain treatments.

## Inosine and Cognition

Very few studies have demonstrated that inosine can increase learning or memory, however, two recent non-clinical studies have shown promising results. The study by Ruhal and Dhingra (2018) used behavioral, biochemical, and histological techniques to demonstrate that inosine can induce learning and memory improvement in aged female rats. Inosine given by intraperitoneal route improved the learning and memory of aged rats in the Morris water maze and elevated plus-maze test. The reduced levels of TNF-α explain these results in the hippocampus and cerebral cortex. In addition, inosine increased the levels of GSH in the hippocampus and the activity of SOD in the cortex and hippocampus, and reduced the level of malondialdehyde, a marker of oxidative stress, in the hippocampus and cerebral cortex (Ruhal and Dhingra, 2018). Furthermore, inosine reduced the degeneration of cells in the CA1 region of the hippocampus, the most critical hippocampal region for memory acquisition, consolidation, and evocation (Ruhal and Dhingra, 2018).

Animal models of Alzheimer’s disease (AD) have also been used to study the effects of inosine. In one study, AD was induced by streptozotocin (STZ), and the animals received inosine by intracerebroventricular route for 25 days (Teixeira et al., 2020). It is well demonstrated that administration of STZ in the brain induces changes similar to those found in AD such as learning and memory deficit, mitochondrial abnormalities, oxidative stress, neuronal cell damage and brain glucose metabolism alterations (Teixeira et al., 2020). This study demonstrated that inosine prevented memory deficits in the inhibitory avoidance task and the Y-maze test (Teixeira et al., 2020). In addition, inosine increased the serum levels of uric acid, a potent natural antioxidant. Teixeira et al. (2020) also demonstrated that inosine prevented an increase in Na^+^/K^+^-ATPase and Mg-ATPase activities and decreased Ca^2+^-ATPase activity in the hippocampus and cerebral cortex induced by STZ. These actions on ion pumps can be related to the antioxidant effects of inosine and neurotransmitter release. Finally, inosine reduced acetylcholinesterase (AChE) activity and increased choline acetyltransferase (ChAT) activity. ChAT is the enzyme responsible for acetylcholine synthesis from choline and acetyl-CoA, and AChE is an enzyme that breaks down acetylcholine. Acetylcholine is the main neurotransmitter involved in memory processes, and studies have shown that changes in ChAT and AChE levels are correlated with cognitive decline in patients with AD (Gutierres et al., 2014; Pacheco et al., 2018). Another study demonstrated that inosine given by intraperitoneal route reduced the neurological severity score and improved non-spatial cognition and memory in mice undergoing a traumatic brain injury model. In the same study, inosine increased the expression of GAP-43, a marker of axonal growth, in the cerebral cortex (Dachir et al., 2014).

Furthermore, inosine effectively reduces the cognitive decline observed in an animal model induced by 12 Gy ionizing irradiation (Hou et al., 2007). Although there are very few studies evaluating the potential of inosine in learning and memory, the data from Ruhal and Dhingra (2018) have shown that inosine has potential and depends, at least in part, on the anti-inflammatory and antioxidative action of this nucleoside. Although these studies did not measure the inosine brain levels, it is clear the potential of inosine to change and treat cognition-related diseases. Then, this topic is an avenue to study and explore the potential therapeutic effects of inosine on cognition.

## Inosine and Parkinson Disease

In the last decade, several *in vitro*, *in vivo*, and clinical studies have demonstrated the potential of inosine to treat Parkinson’s disease (PD). The studies by Cipriani et al. (2010) and McFarland et al. (2013) seem to be the fundamental studies that led to a series of other articles and a large clinical trial that evaluated inosine’s ability to reduce symptoms and the progression of Parkinson’s disease. Cipriani and colleagues showed that urate is an endogenous substance with a high antioxidant capacity. This is the final product of the adenosinergic pathway and is a biomarker of PD progression. McFarland and colleagues demonstrated that urate levels in cortical and striatal tissues tended to be lower in PD and AD than in controls (Cipriani et al., 2010; McFarland et al., 2013). These data strongly suggest that the appearance and development of PD may be related to low urate levels (McFarland et al., 2013; Schwarzschild et al., 2014; Chen et al., 2018). This may be due to the oxidation of dopaminergic cells and other markers in the nigrostriatal region of PD (Church and Ward, 1994; Cipriani et al., 2010). In the SURE-PD (safety urate elevation study - Parkinson disease) clinical trial with a 24-month follow-up, it was demonstrated that the elevation of urate (with serum values between 6 and 8 mg/dL) induced by the administration of inosine was safe. It provides a slower progression on the UPDRS scale, which assesses PD signs and symptoms (Schwarzschild et al., 2014). This effect is attributed to the antioxidant capacity of uric acid, given the relationship between oxidative stress and neuronal death in dopaminergic cells (Cipriani et al., 2014; Schwarzschild et al., 2014; Crotty et al., 2017; Paganoni and Schwarzschild, 2017). This study also demonstrated that these elevated urate levels are safe and do not induce urolithiasis, nephrolithiasis, or cardiovascular changes (Schwarzschild et al., 2014; Chen et al., 2018).

The SURE-PD study also demonstrated that inosine led to a significantly higher increase in urate levels in women than that in men, although women started at a lower baseline level (Schwarzschild et al., 2019). In cerebrospinal fluid (CSF), inosine alone was able to increase urate levels in women. The sex of the patients may have been a factor influencing these distinct changes, as inosine induced a more significant reduction in the UPDRS score in women than that in men, suggesting that high urate levels should be more effective in delaying the progression of PD in women than in men (Schwarzschild et al., 2019). In the SURE-PD study, it was found that oral administration of inosine to patients was able to increase the concentration of ferric reducing antioxidant power (FRAP), a measure of antioxidant capacity, in serum levels but not in CSF. FRAP enhancement is inversely proportional to the rate of clinical decline in patients (Bhattacharyya et al., 2016). In another clinical study in an Asian population, administration of inosine for 1 year did not induce any significant side effects, but there were no clinical improvements on the UPDRS scale (Iwaki et al., 2017).

In a cellular model of PD induced by the addition of H_2_O_2_ to a dopaminergic cells line, called MES 23.5, it was demonstrated that inosine might be a potential treatment for producing anti-inflammatory, trophic, and antitoxic effects *in vitro*. However, these neuroprotective effects of inosine were found only when the MES 23.5 cells were cultured together with a low density of cortical astrocytes, suggesting that this effect is dependent on the release of protective factors from these astrocytes (Cipriani et al., 2014). Another group of researchers evaluated the effect of inosine in an animal model of PD induced by rotenone. In this case, inosine imparted protective effects on protected behavioral, biochemical, and histological parameters. This study suggests that inosine reduced neuroinflammation and oxidative stress due to the suppression of ERK phosphorylation and the downregulation of adenosine A_2__A_ receptor expression (El-Shamarka et al., 2020). In another animal model induced by MPTP, it was demonstrated that the use of an adenosine deaminase inhibitor (ADA), which is the enzyme that converts adenosine to inosine, has a neuroprotective effect. Adenosine A_2__A_ receptor antagonists also have similar effects.

Furthermore, MPTP has been shown to increase ADA activity to induce significantly higher inosine formation. On the other hand, inosine formation after MPTP can also be considered an attempt to reverse the neurodegenerative effects caused by this toxin (Huang et al., 2019). Taken together, these findings confirm the involvement of the adenosinergic pathway in an animal model of PD.

Taken together, even though the inosine results on PD are quite encouraging in the SURE-PD clinical trial and its results corroborate with some preclinical studies, we can conclude that this nucleoside should be more studied by more research groups in both animals and humans to test its real potential to treat this neurodegenerative disease.

## Inosine and Schizophrenia

Several studies have shown that the adenosinergic system may be altered in schizophrenia (SZ), primarily through the effects of adenosine and nucleotide receptors on dopaminergic and glutamatergic signaling, and as deficiency of inosine is associated with SZ (Malewska-Kasprzak et al., 2019). Moreover, many SZ features may be attributed to purinergic signaling dysfunction, called “purinergic hypotheses,” considering that SZ patients present a persistently decreased adenosinergic activity (Lara et al., 2006). Furthermore, adenosine A_2__A_ receptor knockout mice show motor disturbances, social and cognitive alterations, and lateral ventricle enlargement related to psychotic symptoms (Moscoso-Castro et al., 2016). Some studies indicate that inosine is involved in SZ development, but the scarcity of more robust results is still evident.

Using generalized singular value decomposition (GSVD), a new algorithm for metabolomic data analysis, Xiao et al. (2011) assessed metabolomic data from the prefrontal cortex (PFC) of *N*-methyl-D-aspartic acid (NMDA) receptor antagonist phencyclidine (PCP)-treated rats, a translational model of SZ. This approach identified a significant disruption in purinergic reactions, denoted by a substantial increase in adenylosuccinate synthase (ADSS), which is responsible for converting IMP to adenylosuccinate, causing imbalance, an increase in inosine levels and downstream metabolites of other enzymes (e.g., IMP, hypoxanthine, and xanthine).

Stress plays a relevant role in the pathogenesis of SZ. In a refined design, Cai et al. (2017) investigated stress-related metabolic pathways in chronic unpredictable mild stress (CUMS) (stress axis activation), long-term dexamethasone exposure (LTDE) (stress axis activation) rat models, and animals treated with clozapine (CLO), risperidone (RIS), and aripiprazole (ARI). These animals showed an imbalance in the bioenergetic pathways responsible for ATP replenishment in the brain. Compared to controls, inosine levels in the PFC and hippocampus were decreased in the CUMS group and increased in the LTDE and CLO groups. Hypoxanthine and uric acid followed the same pattern in the CUMS and LTDE groups. These findings support the rationale that mitochondrial dysfunction and purine metabolism are closely related to stress-induced pathology. Then, the production of uric acid, a scavenger of reactive oxygen species, is reduced when the adenosine pathway is disturbed because inosine and hypoxanthine are its substrates.

Serum adenosine deaminase (ADA) activity, an enzyme that catalyzes adenosine to inosine, is abnormally high in male SZ patients treated with either typical antipsychotics or CLO, even after adjusting for confounding factors (Brunstein et al., 2007). Conversely, increased gene expression levels of ADA and reduced levels of adenosine transporter, equilibrative nucleoside transporter 1 (ENT1), were observed in enriched populations of pyramidal neurons post-mortem sections from the dorsolateral prefrontal cortex (DLPFC) of patients with SZ. However, no changes in inosine levels were detected in DLPFC tissue homogenates in SZ versus controls (O’Donovan et al., 2018).

Adenosine may play a protective role in SZ, particularly through its antipsychotic effects (Ossowska et al., 2020). Lower ADA activity is linked to a functional variant in the ADA gene (*ADA1^∗^2*), which would raise adenosine levels and, by extension, inosine levels (Battistuzzi et al., 1981). As a result, this polymorphism may protect against SZ development. Indeed, this variant presents a lower frequency in male individuals with SZ in a Brazilian cohort. Patients carrying the G/A genotype demonstrate a decrease in about 20–30% of the ADA’s enzymatic activity (Dutra et al., 2010).

Inosine may also act as guanosine in cellular processes. Inosine is a modified adenosine in RNA, generated by hydrolytic deamination of adenosine and catalyzed by adenosine deaminase acting RNA (ADAR), called A-to-I RNA editing. Deregulation in this biological process affects pathological conditions (Okada et al., 2015). Genome-wide association studies have pointed out that the etiopathogenesis of neuropsychiatric disorders, such as SZ, is related to the host genetic background (Barešić et al., 2019). Searching for possible deregulation of A-to-I RNA editing in frozen human post-mortem brain samples by Silberg et al. (2011), revealed that SZ and suicide victims presented an increased *ADAR* (e.g., *ADAR2*, *ADAR3*, and *ADRB1*) gene variants that codify enzyme isoforms transcripts with decreased catalytic activity. Additionally, *the ADARB1* rs9983925 single nucleotide variant (SNV) has been associated with suicide attempts in Serbian psychiatric patients exposed to traumatic childhood experiences (Karanović et al., 2015).

To the best of our knowledge, no clinical or experimental study has been conducted to evaluate the biological role of inosine in SZ. We have demonstrated that inosine may act through adenosine A_1_, A_2__A_, and A_2__A_ receptors (Lapa et al., 2013; Nascimento et al., 2015). The mitogen-activated protein kinase (MAPK), which regulates inflammation, participates in downstream signaling (Welihinda et al., 2016). Considering that inosine negatively regulates oxidative stress (Ruhal and Dhingra, 2018) and neuroinflammation (Junqueira et al., 2017), all crucial components in the pathophysiological course of SZ, this nucleoside could present relevant positive implications in the treatment of SZ.

## Inosine and Epilepsy

The effects of inosine on epilepsy have been studied since the 1980s. Early studies on this topic indicated that inosine could interact with benzodiazepine receptors, explaining its antiepileptic effects in several animal models of epilepsy, especially tonic-clonic seizures. Recently, a group of studies has suggested that inosine has a pro-epileptic effect, especially in a rodent absence model of human absence epilepsy. Moreover, some studies have demonstrated that inosine brain levels are increased in animal models of epilepsy. This section provides an overview of these findings.

There are many different types of seizures; generalized seizures, that involve large bilateral brain areas, comprehend one of the three main categories of epilepsy (Marshall et al., 2021). Generalized seizures can be presented as tonic-clonic seizures or as absence epilepsy. Tonic-clonic seizures are the most common seizures associated with epilepsy. During the tonic phase, there is loss of consciousness and the body is entirely rigid, and in the clonic phase, there is uncontrolled jerking of the limbs and possible difficulty breathing (Marshall et al., 2021). Absence epilepsy is a brief non-convulsive seizure associated with sudden abruptness in consciousness (Jafarian et al., 2020).

Some studies have indicated that inosine may have antiepileptic effects in different animal models of epilepsy. This evidence emerged in the early 1980s when inosine was identified as one of the main compounds of bovine brain extracts responsible for competitively inhibiting the binding of [^3^H]-diazepam to rat synaptosomal brain membranes (Asano and Spector, 1979). Subsequently, Skolnick et al. (1979) demonstrated that intracerebroventricular (i.c.v.) administration of inosine induced a dose-dependent and time-dependent increase in the latency period to the onset of clonic-tonic convulsions induced by pentylenetetrazol (PTZ). Recently, Ganzella et al. (2011) also demonstrated that i.c.v. administration of inosine protected against quinolinic acid-induced seizures in a time and dose-dependent manner, protecting around 60% of animals at the highest dose tested (400 nmol). Interestingly, intraperitoneal administration of flumazenil, a GABA_A_ receptor antagonist, or caffeine, an adenosine receptor antagonist, did not change inosine protective effect against quinolinic acid-induced seizures (Ganzella et al., 2011). Considering these findings, inosine seems to exert a partial antagonism on PTZ seizures, which can be explained by its interaction with GABA_A_ receptors. Moreover, inosine protects against quinolinic acid-induced seizures in a GABA_A_ receptor-independent manner.

In another approach, i.c.v. administration of inosine in SHR mice 10 min prior to DL-kynurenine significantly reduced the number of mice with DL-kynurenine-induced clonic seizures (Lapin, 1981). Conversely, neither intraperitoneal nor i.c.v. inosine administration prevented seizures induced by PTZ in C57BL/6 or SHR mice (Lapin, 1981).

Systemic administration of inosine has also been shown to have anticonvulsant effects, as demonstrated by pre-treatment with inosine (500–1000 mg/kg), which promoted an increase in the latency of tonic-clonic seizures induced by caffeine in mice and reduced the percentage of animals experiencing seizures (Marangos et al., 1981). In addition, intraperitoneal inosine (1000 mg/kg) reduced the number of mice displaying clonic convulsions and prolonged the latency of clonic convulsions induced by DL-kynurenine C57BL/6 and BALB/c mice (Lapin, 1981). Lewin and Bleck (1985) used BALB/c mice to demonstrate that inosine increased the epileptic dose threshold for PTZ, bicuculline, or picrotoxin and prolonged the time for the first myoclonic contraction induced by these convulsant agents. Interestingly, after subcutaneous administration of the highest dose (1000 mg/kg), an inosine brain concentration of 14.4 μM was reached, suggesting that micromolar concentrations of inosine in the brain are associated with its antiepileptic effects (Lewin and Bleck, 1985).

Recently, Brillatz et al. (2018) demonstrated that inosine also presents antiepileptic effects in a zebrafish epilepsy model with seizures induced by pentylenetetrazol. Interestingly, the authors demonstrated that inosine is the major component of the marine diatom *Skeletonema marinoi*, which also exhibited anticonvulsant effects in zebrafish with PTZ-induced epilepsy (Brillatz et al., 2018). Another research group has proposed a pro-epileptic activity for inosine. Kovács et al. (2015a, b) used Wistar Albino Glaxo/Rijswijk (WAG/Rij) rats, a genetically absent epileptic WAG/Rij rat model that spontaneously generates absence-like seizures. The absence-like seizures in WAG/Rij rats, especially those older than 3 months, can be evidenced in electroencephalographic recordings by bilateral synchronous and spontaneously occurring spike-wave discharges (SWDs) (Kovács et al., 2015a). It has been demonstrated that intraperitoneal inosine injection (500 or 1000 mg/kg) significantly increased SWD in WAG/Rij rats. Furthermore, a combined injection of inosine (500 or 1000 mg/kg) and LPS increased the SWD number and the total time of SWD to a more significant extent than LPS or inosine alone, suggesting that inosine potentiated LPS-induced SWD (Kovács et al., 2015b). Interestingly, muscimol (a GABAa receptor agonist)-induced increase in SWD number was potentiated by inosine (500 mg/kg) (Kovács et al., 2015b). Meanwhile, pre-treatment with bicuculline (a GABAa receptor antagonist) before intraperitoneal inosine administration (500 mg/kg) seems to prevent inosine-induced SWD (Kovács et al., 2015a). Taken together, these findings suggest that inosine induces absence-like seizures in WAG/Rij rats, and GABAa receptors could mediate inosine effects. In a different approach, Lakátos et al. (2018) demonstrated that the combined application of allopurinol (a xanthine oxidase inhibitor) and inosine significantly increased the SWD number when compared to allopurinol treatment or inosine treatment. These findings suggest that an increase in the endogenous levels of inosine due to the allopurinol inhibition of the xanthine oxidase enzyme may contribute to absence-like seizures in WAG/Rij rats, reinforcing the possible pro-epileptic effects of inosine in absence seizures (Lakátos et al., 2018).

Some studies have been designed to study inosine brain levels in animal models of epilepsy. Lewin and Bleck (1981) demonstrated that a sub-convulsive series of electroshocks in mice induces clonic movements and tonic extension of the hindlimbs. In this electroshock-induced seizure model, the authors showed that inosine brain levels increased significantly, reaching their maximal value at 5 min (for a 60-Hz stimulus) and 1 min (for a 3-Hz stimulus). Interestingly, phenytoin or phenobarbital administration markedly reduced the increase in inosine brain levels induced by electroshock (Lewin and Bleck, 1981). These results suggest that an early low increase in inosine brain levels following electroshock play a role in seizure generation and propagation. However, high concentrations of inosine after epileptic seizure recovery may contribute to seizure termination (Lewin and Bleck, 1981). In addition, Lehmann (1987) demonstrated that when folate was bilaterally injected into the amygdala of Albino rabbits, it induced limbic seizures and produced an increase in the hippocampal levels of inosine 10–130 min after injection. The authors suggested that increased inosine levels could be associated with ATP catabolism and indicate a minor perturbation of the cerebral energy state due to epileptic seizures induced by folate (Lehmann, 1987). In contrast, inosine levels in the cerebrospinal fluid (CSF) of patients with a rare disease characterized by progressive myoclonus epilepsy did not differ significantly from inosine levels in the CSF of control patients (Ohisalo et al., 1983). In an interesting case report, Ito et al. described a 4-year-old girl diagnosed with chronic mumps virus infection that presented with generalized tonic-clonic seizures. It was demonstrated that the attacks subsided gradually after administering inosine pranobex (isoprinosine) 100 mg/kg/day (Ito et al., 1991). A marked decrease was observed in slow-wave activity by EEG after approximately 1 month of treatment with isoprinosine, and the patient became seizure-free 9 months after onset (Ito et al., 1991). However, the authors did not rule out the possibility of spontaneous remission (Ito et al., 1991). In addition, considering that inosine pranobex is a combination of inosine, acetamidobenzoic acid, and dimethylaminoisopropanol, it is impossible to conclude the actual contribution of inosine for the improvement of the epileptic seizures presented by the patient.

Considering these findings, there is an evident correlation between inosine and epilepsy. However, further studies are needed to clarify the nature of the aforementioned correlation, since some studies suggest a possible pro-epileptic effect, especially regarding absence seizures. In contrast, others have suggested an antiepileptic effect, mainly in tonic-clonic episodes. In this sense, it would be interesting to evaluate inosine effects in animal models of chronic epilepsy and models used to identify drugs with efficacy against pharmacoresistant seizures. In parallel, identifying the mechanisms involved in the inosine effects in epilepsy is critical and can be performed using genetic, pharmacological, and *in vitro* approaches. Moreover, considering that some studies have identified increased inosine levels in the brains of animals subjected to epilepsy models, it would be essential to clarify whether these inosine levels contribute to the generation/propagation or termination of epileptic seizures. Together, these studies could extend the knowledge regarding inosine in epilepsy.

## Inosine and Anxiety

The interaction between inosine and anxiety is not entirely understood. Most studies dealing with this issue have been published in the late 1970s and early 1980s (Asano and Spector, 1979; Slater and Longman, 1979; Paul et al., 1980; Bold et al., 1985). Most of these studies used *in vitro* experiments and suggest a possible interaction between inosine and benzodiazepine receptors. However, few studies have evaluated the *in vivo* effects of inosine on anxiety. In this section, a review of these studies and suggestions for future directions will be provided to better understand the role of inosine in anxiety.

Identification of specific receptors in the brain that mediate the anxiolytic, muscle relaxation, and hypnotic effects of diazepam led to the hypothesis that an endogenous ligand for benzodiazepine receptors must also exist in the brain. In this context, Asano and Spector (1979) identified inosine present in bovine brain crude extracts as an endogenous competitive inhibitor of [^3^H] diazepam-binding benzodiazepine receptors. Inosine inhibited the binding of about 50% of [^3^H] diazepam at a concentration of 1.5 mM to the benzodiazepine receptor in the rat brain (Asano and Spector, 1979). Regarding tissue specificity, the authors demonstrated that inosine had a much lower affinity for benzodiazepine receptors in peripheral tissues, such as the kidney and liver, than for those in the brain (Asano and Spector, 1979). Similar results were found previously (Paul et al., 1980) and later (Skolnick et al., 1978), providing evidence that inosine is a compound from brain extracts that competitively inhibits [^3^H] diazepam and [^3^H] flunitrazepam (Yarom et al., 1998) binding to the synaptosomal membrane. In addition, inosine inhibited neuronal excitability by increasing membrane conductance in cultured spinal neurons, an effect that was blocked by flurazepam (MacDonald et al., 1979). Together, these findings suggest that inosine could play a neuromodulatory role, mimicking or antagonizing the pharmacological effects of benzodiazepines. However, functional studies such as patch clamp studies or ion flux studies in cells transfected with the benzodiazepine receptors should have been conducted to clarify if inosine acts as an antagonist, agonist or even an inverse agonist of benzodiazepine receptors.

Interestingly, Slater and Longman (1979) used an *in vivo* method to detect drugs with GABA-mimetic properties. In this study, diazepam was injected striatally into the globus pallidus and a highly significant slowing of the head-turn, a behavior that can be modulated by GABA agonists or antagonists, was observed. This behavior was used to demonstrate the *in vivo* GABA-mimetic properties of benzodiazepines (Crossman et al., 1977, 1979). Intrapallidal injection of inosine (10 μg) had no significant effect on the head turn (Slater and Longman, 1979). However, when injected either before or together with diazepam, inosine completely prevented diazepam-induced head-turn slowing (Slater and Longman, 1979). These results suggest that inosine has no GABA-mimetic or benzodiazepine-like properties when tested with the head-turn model.

In contrast, inosine seems to interact with benzodiazepine receptors and can antagonize diazepam actions in the globus pallidus (Slater and Longman, 1979). These findings suggest that inosine could have an anxiogenic effect by antagonizing the actions of diazepam. In this context, Bold et al. (1985) demonstrated that inosine inhibited [^3^H]-flunitrazepam binding to the benzodiazepine receptor in rat cerebellar cell membranes, with an IC_50_ value of 8 mM and a low affinity for the receptor. Moreover, the same study used the social interaction test in rats, which has been proposed as a valid test for measuring anxiety. In this test, inosine did not increase social interaction in a manner comparable to that of benzodiazepines (Bold et al., 1985), indicating that inosine did not present an anxiolytic effect in the social interaction test. The authors did not evaluate whether inosine was able to reduce social interactions. It was also not assessed if inosine was able to inhibit the benzodiazepine-induced increase in social interaction. Thus, no conclusion could be drawn regarding the possible anxiogenic effects of inosine. Crawley et al. (1981) used an apparatus that records the number of transitions made by a mouse between an illuminated open-field compartment and a dark enclosed compartment. Benzodiazepine administration prior to the test significantly increased the number of such transitions and could be inferred to be related to the occurrence of an antianxiety effect.

Interestingly, inosine at doses ranging from 10 to 300 mg/kg (below the sedative range, 500–1000 mg/kg) reversed the diazepam-induced increase in the number of transitions between light and dark compartments, suggesting that inosine can antagonize the anxiety-reducing actions of benzodiazepines (Crawley et al., 1981). In corroborating this finding, Wagner and Katz (1983) demonstrated that synthetic purines such as 2′-deoxyinosine and 7-methylinosine, that bind to benzodiazepine receptor, are structurally similar to inosine and seem to present a dose-related anxiogenic effect in Sprague-Dawley rats (Wagner and Katz, 1983). These results reinforce the findings, suggesting that endogenous inosine shows anxiogenic activity due to its interaction with benzodiazepine receptors.

In contrast to most of the findings that suggest a possible anxiogenic effect of inosine, Okuyama et al. (1998) demonstrated that inosine (10–60 mg/kg) administered subcutaneously to male ddY mice (an outbred mouse strain derived from a mouse colony at the Institute of Infectious Diseases of Tokyo University) Yamazaki et al. (2012) presented an anxiolytic-like effect in the Vogel-type anti-conflict test. Interestingly, in this study, inosine and other purines such as adenosine, AMP, c-AMP, and adenine have been isolated from the fruit of *Euphoria longana* (Longan Arillus); however, only inosine and adenosine seemed to have an anxiolytic-like effect (Okuyama et al., 1998). The Vogel-type anti-conflict test presents an excellent predictive value for classical anxiolytic drugs, such as benzodiazepines (Campos et al., 2013). However, the test also responds to non-anxiolytic drugs (Campos et al., 2013), producing false-positive results. It is important to mention that the potential antinociceptive properties of inosine (Nascimento et al., 2010, 2015; Macedo-Junior et al., 2013) could influence the result found by Okuyama et al. (1998), considering the Vogel test involves the response of mice to a noxious electrical stimulus employed as punishment.

According to the findings described here, inosine seems to interact with benzodiazepine receptors, as suggested by studies that used cell membrane binding assays. However, these findings are relatively old, dating back to the late 1970s and throughout the 1980s. Taking that into account, it is crucial to confirm whether inosine acts as a benzodiazepine receptor antagonist. In these sense, functional studies in cells transfected with benzodiazepine receptors and/or in primary cell cultures could not only ensure the interaction of inosine with benzodiazepine receptors but also in particular demonstrate whether this interaction would be able to modify GABA channels functioning in the cell activation/inhibition dynamics (e.g., using patch-clamp assays). Another critical point is to understand whether inosine can change the activity/expression/levels of enzymes, proteins, or second messengers related to the benzodiazepine receptor signaling pathway and whether these findings could be related to its anxiolytic or anxiogenic effects. In addition, the assessment of the anxiolytic or anxiogenic activity of inosine should be performed on animal models associated with the subjacent neurobiology and which meet the criteria of predictive, face, and construct validation. Together, these findings clarify the role of inosine in anxiety.

## Inosine and Depression

Several studies have demonstrated a relationship between inosine and depression. These studies showed the effect of inosine treatment in different animal models of depression and paved the way to identify the mechanisms of inosine action in depression. Interestingly, clinical studies have shown that serum inosine levels are associated with major depressive disorders in humans. A review of these studies will be provided in this section, and future directions will be suggested to incorporate inosine in treatment regimens or as a diagnostic biomarker in depressive disorders.

In our database search, the study by Kaster et al. (2013) seems to be among the first to evaluate the possible antidepressant effect of inosine in animal models of depression. In this study, the authors first performed a dose-response curve for inosine in the force swim test (FST) and the tail suspension test (TST). The results demonstrated that inosine significantly reduced the depression behavior in both tests. Similarly, Gonçalves et al. (2017a) demonstrated that inosine given by intraperitoneal route presented antidepressant activity in the TST. Corroborating these findings, Yuan et al. (2018) demonstrated that inosine presented an antidepressant-like effect in the FST in rats. In an interesting approach, an inosine-supplemented diet and an inosine-supplemented tap water presented an antidepressant-like activity, preventing, respectively, chronic unpredictable stress and chronic social defeat stress-induced in set of depression-like behavior in mice (Muto et al., 2014). In addition, inosine improves neuronal proliferation in the mouse brain and promotes neuronal viability and neurite outgrowth in cultured neocortical neurons (Muto et al., 2014).

Regarding action mechanisms related to inosine antidepressant effects, Kaster et al. (2013) demonstrated that both adenosine A_1_ receptor antagonist (DPCPX) and adenosine A_2__A_ receptor antagonist (ZM241385) were able to prevent inosine antidepressant-like effects. Moreover, activation of PKA, PI3K/Akt, ERK1/2, CaMKII and mTORC1 and the inhibition of GSK-3β and NMDA receptors seems to be involved in the inosine antidepressant-like effects in the TST (Gonçalves et al., 2017a,b). Furthermore, hippocampal CREB phosphorylation, MAPK phosphorylation and BDNF transcription increased 24 h after a single intraperitoneal administration of inosine (Muto et al., 2014; Gonçalves et al., 2017a). Similarly, Yuan et al. (2018) demonstrated that inosine (10 or 50 mg/kg, i.p.) previously administered at 30 min, 6 h, and 24 h in rats induced a significant increase in phosphorylated ERK and CREB in the rat hippocampus.

From a clinical point of view, it has been suggested that the plasma levels of inosine are significantly reduced in individuals (aged 20–71 years) with major depressive disorder (MDD) when compared with control non-depressive individuals (Ali-Sisto et al., 2016). However, the authors did not observe any statistically significant difference in inosine plasma levels between the remitted and non-remitted groups (Ali-Sisto et al., 2016). On the other hand, Mocking et al. (2021) demonstrated that inosine plasma levels were decreased in both males and females with recurrent major depressive disorder in remission. Zhou et al. (2019) studied the potential biomarkers of depression in children and adolescents with MDD. The authors found that inosine plasma levels are reduced in children and adolescents diagnosed with MDD when compared to health controls. Interestingly, regression analyses suggest that inosine plasma levels are more pronounced in the plasma of boys and among individuals with more severe symptoms of depression (Zhou et al., 2019). Furthermore, inosine plasma levels were significantly higher in individuals treated with antidepressant drugs than in healthy individuals. These studies suggest that inosine could be used as a diagnostic biomarker in individuals with MDD, especially in children and adolescents.

The evidence presented here suggests that inosine induced an antidepressant-like effect in various animal models of depression. However, it is important to note that most studies that evaluated inosine effects in animal models meet only the predictive validity criterion, such as FST and TST. In this sense, it would be essential to extend the test with inosine to animal models that also meet construct and face validities to evaluate its effect on the molecular and biochemical CNS alterations present in depression and classic depression symptoms like anhedonia. It would also be interesting to assess whether inosine increases serotonin/norepinephrine levels in the CNS in animal models of depression. Together, the investigation of these points could confirm, clarify, and extend the role of inosine in depression.

## Inosine and Neural Regeneration

The therapeutic effects of inosine, which regulates neuron activity, have been described in literature by several research groups. In the last four decades, studies have demonstrated that inosine can be released by cultured neuronal cells, modulate the neurotransmitter release, and activate intracellular signaling pathways that regulate the expression of multiple genes involved in axon outgrowth *in vitro* and *in vivo*. One of the first studies to show that inosine can modulate neuronal activity *in vitro* in adrenergic neurons has been the study by Zurn and Do (1988). The involvement of inosine in other modulatory events, including the growth and regeneration of neuronal connections, was investigated in cultured neural goldfish retinal ganglion cells and rat retinal ganglion cells (Benowitz et al., 1998; Petrausch et al., 2000). These studies have described inosine-stimulating axonal outgrowth through an intracellular mechanism that activates protein kinase–N, GAP-43, protein T alpha-1 tubulin, and L1, a cell adhesion molecule (Benowitz et al., 1998; Petrausch et al., 2000; Cook et al., 2004), mimicking at least one aspect of the molecular changes that underlie axonal regeneration *in vivo*.

These results have led researchers to perform confirmatory *in vivo* studies using animal models, including sciatic nerve injury (Hadlock et al., 1999; Cardoso et al., 2019), controlled cortical impaction (CCI) injury (Smith et al., 2007), closed head injury (Dachir et al., 2014), spinal cord injury (SCI) (Bohnert et al., 2007; Kim et al., 2013; Chung et al., 2015), and ischemic stroke (Chen et al., 2002; Zai et al., 2009, 2011; Benowitz and Carmichael, 2010). In the first published studies investigating the pharmacological effects of inosine, inosine was administered directly into the CNS using osmotic minipumps. The results obtained from these studies corroborate previous *in vitro* data. It has been demonstrated that inosine can stimulate undamaged neurons to extend collateral branches that grow into areas of the brainstem and spinal cord that lose their normal innervation due to injury when administered alone (Hadlock et al., 1999) or together with oscillating field stimulation (OFS) treatment (Bohnert et al., 2007). In addition, inosine combined with NEP1-40, a potent antagonist of the Nogo receptor (the receptor of Nogo-A, a myelin-associated glycoprotein considered to be an inhibitor of neurite growth), doubled the number of axon branches extending from neurons in the intact hemisphere and induced the growth of bouton-like structures in the gray matter into the denervated side of the spinal cord (Zai et al., 2011).

Kim et al. (2013) also demonstrated promising effects of inosine administration on neuron outgrowth. In 2013, inosine was administered intravenously, showing that systemic administration induces axon sprouting in the CNS and increasing serotonergic input to the lumbar spinal cord, indicating that inosine can cross the blood-brain barrier. Thus, in subsequent studies, inosine effects were investigated systemically by intraperitoneal, intravenous, and oral routes in several animal models (Kim et al., 2013; Dachir et al., 2014; Chung et al., 2015; Moore et al., 2016; Cardoso et al., 2019). In these studies, inosine reduced the loss of urinary tract function (Chung et al., 2015), decreased the neurological severity score, and improved motor function (Dachir et al., 2014; Cardoso et al., 2019). All these effects are related to the induction of axon regeneration. Interestingly, when inosine is administered daily via the oral route (500 mg/kg) mixed in flavored yogurt, it induces a more remarkable recovery in terms of a return to standard grasp patterns (finger-thumb pinch) in a cortical injury model (Moore et al., 2016).

Evidence has indicated that the motor improvement observed after inosine treatment *in vivo* was directly related to an increase in the expression of growth-associated membrane phosphoprotein GAP- 43 in neural cells, suggesting that inosine may act similarly *in vivo* and *in vitro*, inducing a program of gene expression that enables axon regeneration. Another protein, Mst3b, is related to the inosine mechanism *in vitro*, and is classified as a purine-sensitive protein kinase that plays a crucial role in axon outgrowth (Irwin et al., 2006).

In addition to a study that revealed a purine-sensitive protein, two exciting studies using an animal model of ischemic stroke and gene microarray analysis have demonstrated that inosine can affect gene expression in the CNS. The genes that are selectively upregulated by inosine include those encoding tissue inhibitors of metalloproteinase (*timp1*), metallothionein, galectin 3, and complement cascade proteins (Zai et al., 2009). Moreover, genes involved in cell signaling, cell morphology, cell maintenance, assembly and organization, DNA replication, recombination and repair, and nervous system development and function could be upregulated. The authors suggest that chromatin remodeling and genome-wide transcriptional changes may depend on Mst3b activation, a hypothesis that requires further testing (Zai et al., 2011). The data demonstrating inosine effects activating specific proteins and modulating gene transcription are quite promising and may be linked to the enhanced ability of neurons to modify synaptic relationships or promote axon branching. This induction of neural plasticity and axonal sprouting is the basis for behavioral improvement.

Besides the mechanisms involved in inosine-induced plasticity that have been investigated at the molecular level, the contribution of adenosine P1 receptor-signaling pathways to neuronal outgrowth needs to be addressed, since its role in inflammation, immunity, pain, and CNS pathologies has been reported in the literature. The inosine-induced neuronal outgrowth observed in multiple animal models of neurological injury, its clinical safety in humans, and the feasibility of administration, including the oral route, indicates inosine as an excellent candidate for clinical trials of treatment regimens of patients with CNS injuries.

## Future Advances and Perspectives

A few decades ago, inosine was considered an inert molecule resulting from the degradation of adenosine. In this review, we discuss the current knowledge and describe the myriad scientific evidence that highlight the critical effects of inosine on the central nervous system. Evidence of inosine-binding sites, mainly adenosine receptors, supports an increment of inosine research in the future investigating its physiological role in the CNS disorders and its potential for the development of new therapeutic approaches to treat neurological and psychiatric disorders (see Figure 1). We believe that inosine is a great molecule that has been neglected until now and that it is able to treat several physiological disturbs safely. Therefore, we hope that this review can support and promote a path for further investigation of the benefits of inosine through preclinical (*in silico*, *in vitro*, and *in vivo*) and clinical studies.

## Author Contributions

FN planned and was responsible for the coordination and overall supervision of the study. All the authors contributed equally to writing and editing the manuscript and approved the final manuscript.

## Conflict of Interest

The authors declare that the research was conducted in the absence of any commercial or financial relationships that could be construed as a potential conflict of interest.

## Publisher’s Note

All claims expressed in this article are solely those of the authors and do not necessarily represent those of their affiliated organizations, or those of the publisher, the editors and the reviewers. Any product that may be evaluated in this article, or claim that may be made by its manufacturer, is not guaranteed or endorsed by the publisher.
